# Electrified Nanogaps under an AC Field: A Molecular Dynamics Study

**DOI:** 10.1021/acs.jpcc.4c05105

**Published:** 2024-11-29

**Authors:** Mahdi Tavakol, Alexander Newbold, Kislon Voïtchovsky

**Affiliations:** Physics Department, Durham University, Durham DH1 3LE, U.K.

## Abstract

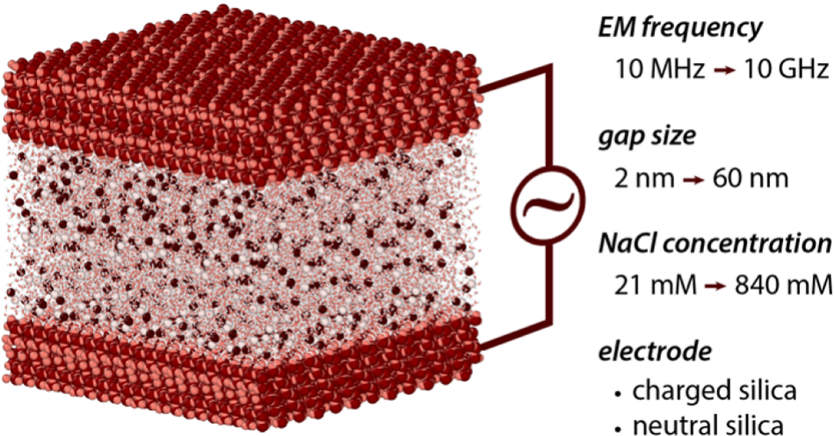

The organization and dynamics of ions and water molecules at electrified solid–liquid interfaces are generally well understood under static fields, especially for macroscopic electrochemical systems. In contrast, studies involving alternating (AC) fields tend to be more challenging. In nanoscale systems, added complexity can arise from interfacial interactions and the need to consider ions and molecules explicitly. Here we use molecular dynamics (MD) simulations to investigate the behavior of NaCl aqueous solutions at different concentrations confined in nanogaps under AC fields ranging from 10 MHz to 10 GHz. We explore the impact of the gap size (2–60 nm) and of the solid material composing the electrode (silica, charged silica, or gold). Analysis of the transient and stable responses of the system shows that the total transverse dipole *M*_*z*,total_ formed by the water molecules and the ions across the gap is always able to counter the applied field regardless of AC frequency, NaCl concentration, or electrode material. As expected, the ions lag at higher frequencies, leading to a capacitive behavior. This effect is fully compensated by water dipoles that lead the field, reaching a maximum lead at a specific frequency which depends on salt concentration and gap size. Changing the gap size affects the magnitude of *M*_*z*,total_. Finally, the electrode material is shown to affect the electrolyte behavior in the gap region. We anticipate these results to be useful for nanoscale dielectric spectroscopy, including scanning probes.

## 1 Introduction

Electrified interfaces between solids and aqueous electrolytes are common in many systems, from biological membranes under their natural transmembrane potential^1^ to sensing devices^2,3^ and in the development of fuel cells and energy storage solutions.^4,5^ The behavior of these interfaces is generally well understood at the macroscale and obeys established continuum theories such as the Gouy–Chapman–Stern model^6−8^ that rely on the Poisson–Boltzmann formalism;^8−10^ counterions accumulate at the surface of the immersed solid to counter the surface potential, creating an electrical double layer (EDL) whose thickness is characterized by its so-called Debye length,^9^ λ_D_. In the case of time-varying electrical potentials, the Boltzmann formalism can be augmented to take into account the ionic diffusion in response to the changing field: the Poisson–Nernst–Plank formalism.^11^ Both Poisson–Boltzmann and Poisson–Nernst–Plank have proven to be extremely successful to quantitatively describe the behavior of ions at charged or electrified interfaces, often down to the nanometer scale. However, to understand the behavior of the interface at the molecular level, it is necessary to also consider the finite size of the water molecules and hydrated ions and the local orientation of the molecular dipoles. This is implicitly taken into account in continuum models through *effective* values, but gaining molecular-level insights of the organization and dynamics at the interface remains challenging. This is particularly true for nano-confined systems where the interface can dominate the system not only through electrical interactions but also via chemical interactions. Additionally, at high enough frequencies, the interplay between the dynamics of water molecules and ions becomes nontrivial due to water dipoles reacting far quicker than diffusing ions.^3,12^

Generally, the response of a given system to an AC field is investigated by use of dielectric spectroscopy^12,13^ to track processes ranging from the diffusion motion of macromolecules or calcium ions between electrical synapses^14^ to the reorientation of liquid’s molecular dipoles,^12^ electronic motion in perovskite voltaic cells,^15^ and ultrafast electronic phenomena in the visible or UV spectrum beyond 10^16^ Hz. Specific molecular or electronic processes can be quantified through the changes in the field amplitude and phase they induce at given frequencies:^13^ when the frequency applied field is too fast for allowing charges of dipoles to follow, a transition can be observed. Probing the response of ions and molecular dipoles in an electrolyte can usually be achieved electrically, with the relevant frequencies typically in the radio domain (MHz to GHz). Experimentally, this is often referred to as electrochemical impedance spectroscopy^14^ (EIS).

With the motion of ions being diffusion limited in an electrolyte, the response of the system to EIS measurements tends to become highly sensitive to the local geometry and the chemical composition of the electrodes when at the nanoscale.^3^ This can be exploited for controlling the flow of an electrolyte by AC field in nanofluidics systems^16,17^ or in the development of single-molecule nanosensors.^2,18^ Separately, experimental strategies are being devised to quantify the dipolar behavior and charge mobility of electrolytes near electrodes with nanoscale spatial precision^19−26^ to help with the current need for new and cleaner energy storage and production solutions. This is typically achieved by combining aspects of dielectric spectroscopy or EIS with scanning probe techniques to achieve suitable spatial resolution.^19−21,23,27^ Significant advances have been achieved,^28,29^ but interpretation of experimental results is often only possible through complementary computer simulations to disentangle molecular interactions from confinement effects and the electrical response of the system.

In this study, we develop a molecular dynamics (MD) simulation framework to investigate the molecular dynamics of aqueous solutions containing different concentrations of NaCl when exposed to AC electric fields inside nanogaps. The range of frequencies probed explores the typical relevant EIS domain for ion motion and water dipole reorganization (10 MHz to 10 GHz), and the solid material composing the edges of the gap is made of neutral silica, a common blocking electrode in scanning probe measurements.^19,23,27^ We also investigate charged silica and gold (without explicitly modeling electronic interactions) as confining solids and systematically explore the impact of a gap size from 2 to 60 nm while keeping the amplitude of the applied voltage constant to reflect common experimental conditions. The goal of the study is to provide molecular-level insights into the interplay between interfacial chemical interfaces and electrostatic interactions across the range of frequencies and geometries typical to nano-EIS systems, especially for scanning probe measurements.

## 2 Methods for the Simulations

### 2.1 Setup of the System

**Figure 1:**
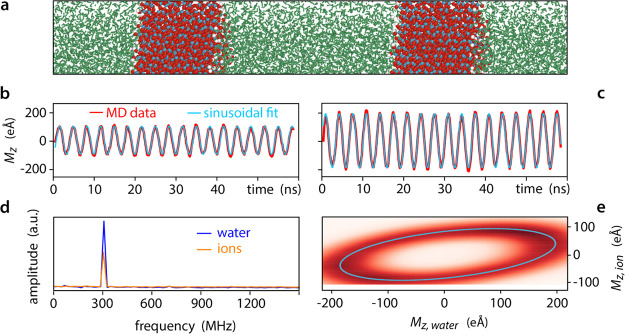
Simulation setup and principles used for data analysis. A typical simulation box (a) comprises two solid slabs (here silica with atoms in red and blue) and the solution (water molecules shown in green). From MD, the transient response of the ions (b) and water (c) to the applied AC potential (red) can be well approximated with a single sinusoid (cyan). In practice, it is the total net dipole *M*_*z*_ for all of the ions and water molecules between the slabs that is being measured. Fourier analysis of the MD transients (d) confirms that the single frequency (the driving AC frequency, here 300 MHz) is a suitable approximation for the analysis. This can further be visualized in two dimensions when plotting the net dipole obtained for the ions (*M*_*z*,ion_) against the net dipole for water (*M*_*z*,water_) (e). The results of 10 simulation runs are condensed in a single graph (red dots) with the Fourier approximation appearing as a the blue ellipse. The circularity of the ellipse represents the magnitude of the ratio *M*_*z*,ion_/*M*_*z*,water_, whereas the ellipse orientation is determined by the difference in the phase lag of each contribution toward screening the driving field.

The MD simulations are run on a system composed of two solid slabs separated by a distance of *d* and filled with an aqueous saline solution (Figure 1a). The system comprises either noncharged silica slabs or one charged silica slab and one gold slab. The dimensions of the system are 3.83 × 3.92 nm^2^ for the *xy* plane and 19.38 nm in the *z* direction. The silica coordinates were taken from the Q3 surface of the interface force field database.^30^ The neutral silica was set up with any SiO^–^Na^+^ surface groups, whereas the charged silica contained 0.67 SiO^–^Na^+^ groups per nm^2^ (assuming a neutral pH of 7). Both types of silica slabs have a 2.3 nm thickness and contain 4.7 silanol groups per nm^2^. The computed surface charge density for the charged silica slab corresponds to that of silica at pH 7.^31^ The gold slab has a thickness of 1.36 nm with the 111 plane facing the solution. The interface force field^32^ was chosen for modeling the interatomic interactions involving the silica and gold atoms, together with the flexible SPC/Fw model for water molecules.^33^ The self-diffusion coefficient of SPC/Fw is in agreement with experimental measured values.^34^ Additionally, preliminary results for the ion distribution near a charge silica surface showed no appreciable difference between the free energy for counterion adsorption derived with the water models of H_2_O/DC, TIP 3p-ST, and SPC/Fw. The dimensions of the simulation box dimensions were chosen to ensure that the same distance is held between slabs (∼7.3 nm for fixed distances) either directly or through periodic boundary conditions. The simulation box was filled with 8000 SPC/Fw water molecules, unless stated otherwise, and the required amounts of Na^+^ and Cl^–^ were added to reach the desired salt concentration (see details in Table S1). We report both the total transverse electrical dipole moment *M* formed by the water molecules and the ions located in the gap, and the values of *M* scaled by the ratio of gap size to our reference value of 6.88 nm. The scaling effectively offers a form a normalization by the number of water molecules/ions present in the system so as to provide an average value per water molecule/ion and aid comparison.

Every simulation followed several consecutive stages. After an initial energy minimization to remove bad contacts, an equilibration stage of 10 ns was implemented, during which no electric field was applied to the system. Subsequently, an AC electric field with the desired frequency (ranging from 10 MHz to 10 GHz) was applied to the system following a method described elsewhere.^35^ In short, the external voltage was applied between the slabs through an imposed electric field as described by Gumbart et al.^36^ In this approach, the value of the imposed field *E* at any time is such that it creates a potential difference of Δ*V* across the box size with *E* = Δ*V*/*d*. Previous simulations^35^ showed that *d* must be taken as the closest distance between the slab’s surfaces to set a voltage difference of Δ*V* across the solution located inside the gap region. The electric field was applied to the whole simulation box. The magnitude of the applied AC voltage was 14.14 V (10 V root mean square), resulting in a maximum field of 0.673 V/Å across the box. The magnitude of the voltage is motivated by previous works^35,37^ where similar voltages could reproduce experiment results^37^ and reveal the physical mechanisms governing the organization and dynamics of the ions at the interface (for more information, please see ref (31)). A Nosé–Hoover thermostat and barostat were used to control the temperature and pressure at 300 K and 1 bar, respectively.^38^ MD simulations were done in the LAMMPS software^39^ (version of 23 Jun 2022). The Matplotlib package of Python^40^ was deployed alongside the OVITO software^41^ for visualization purposes. Several Python and C++ codes were written to analyze the dipole moment of water molecules and ions in the system. In the current study, 65 different simulations were done for durations in the range of 20–150 ns, each repeated between 2 and 10 times, with a cumulated simulation time of 10.09 μs (more details in Table S1).

### 2.2 Analysis of the Data

The total transverse electrical dipole moment *M*_*z*_ formed by the water molecules and the ions located inside the gap was calculated for each step of the simulations. *M*_*z*_ is a useful proxy for distinguishing the response of water (*M*_*z*,water_) and ions (*M*_*z*,ion_) to the applied AC voltage, measured along the direction of the electric field (Figure 1b,c). Both transients show a stable and periodic response, making them suitable for Fourier analysis with a single frequency (the driving frequency) needed for approximating each transient (Figure 1d,e). This was confirmed by repeating some of the simulations 10 times, obtaining a stable response well-described by a single sinusoid for each water and ion (Figure 1e). In principle, statistical noise and lack of ergodicity limit the minimal number of simulations needed to reach acceptable reproducibility. However, separate analysis for water and ions showed that two simulations are already enough with no significant subsequent evolution of the result.

For the ion dipole moment of the system with gold-charged silica slabs, Fourier analysis revealed a component with a frequency of zero in addition to the expected component at the AC driving voltage frequency. This DC component represents the response of the system to the additional static screening of silica surface charges by ions in the solution.

The ion and water density profiles over the whole simulation were obtained through a moving average coded in Python. An averaging window width of 1 Å was used with a total of 1000 windows evenly distributed across the simulation box unless stated otherwise.

For the Fourier series analysis, a C++ code was written. The phase in the *M* response was defined as φ_0_ in the equation *M* = sin(ω*t* + φ_0_).

The dielectric constant in MD simulation is obtained through eq 1 in which *M*, ε_0_, *V*_w_, *k*, *T*, ⟨ ⟩, and ε stand for the total water dipole, vacuum permittivity, water volume, Boltzmann’s constant, temperature, ensemble average, and dielectric constant, respectively. Because the outcome of running 10 simulations showed that *M*_*z*,water_ becomes closer to its Fourier series with increasing number of simulations, the dielectric constant equation was modified to eq 2 in which the *M*_*x*_, *M*_*y*_, and *M*_*z,*Fourier_ represent the *x*, *y,* and the Fourier series of the *z* component of water molecules.
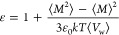
1
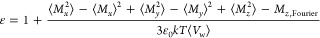
2

## 3 Results

### 3.1 The Transient Response

**Figure 2:**
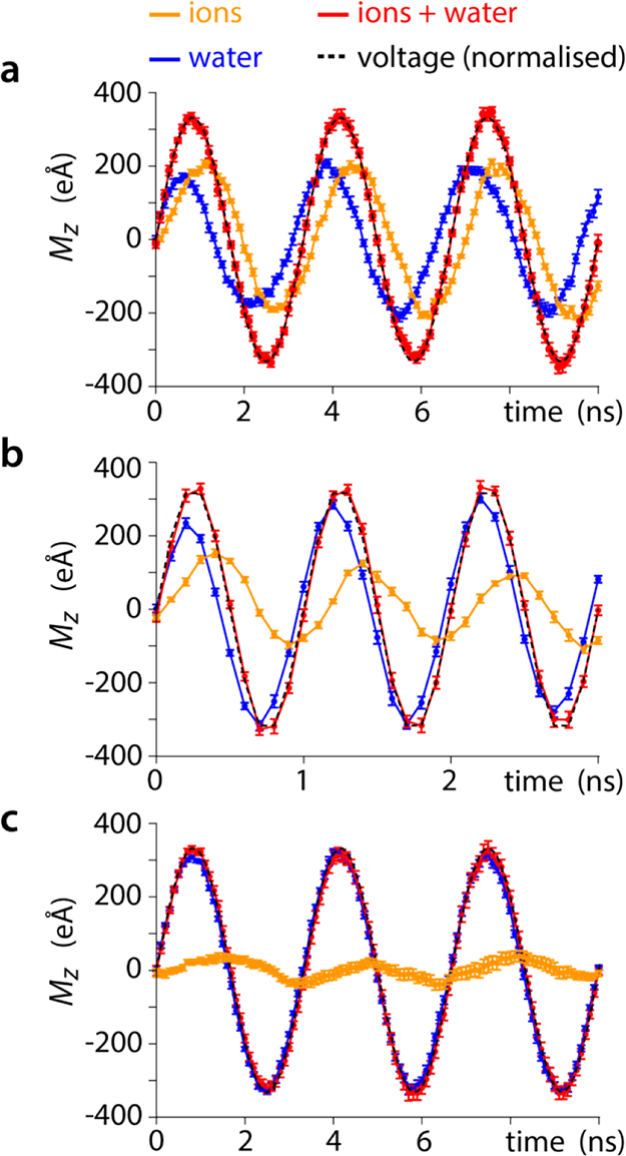
Initial response of ions and water inside the uncharged silica nanogap to an applied AC voltage. As the voltage is initially applied (time zero), the resulting electric field triggers an initial response from the ions and the water molecules (transient), rapidly converging to a steady state over less than half a cycle. The impact of the AC frequency and ionic concentration is investigated for (a) 300 MHz and 210 mM NaCl, (b) 1000 MHz and 210 mM NaCl, and (c) 300 MHz and 21 mM NaCl. In all cases, the net instantaneous dipoles resulting from the ions and the water are given separately, together with the sum of their effect and the voltage driving the response (normalized to *M*_*z*,total_ to aid readability). Note that *M*_*z*,total_ represents the vectorial sum of *M*_*z*,ion_ and *M*_*z*,water_.

The transient response of the water and ions to the imposed field is shown in Figure 2 for the uncharged silica–silica system, with an interslab distance of 6.88 nm and different salt concentrations and under several AC frequencies. Similarly to Figure 1, an average of 10 different simulation runs was used. From starting the field at time zero, the transient response of the system is typically visible over the first AC cycle, rapidly converging toward a steady, periodic response.

The total transient response, *M*_*z*,total_, of the solution is always in phase with the applied AC voltage and cancels out most of the applied field. This second point was verified with simulations conducted over the same system but applying a DC voltage of magnitude identical to the amplitude of the AC voltage used here (14.14 V). The DC simulation was run over 50 ns, yielding a constant value of *M*_*z*,total_ = 334 ± 22 eÅ within error (Figure S1).

Although the magnitude of *M*_*z*,ions_ and *M*_*z*,water_ changes when evolving from a transient to stable regime, the amplitude of *M*_*z*,total_ remains unchanged (Figure 2) and with a magnitude identical to the value obtained in DC simulations (Figure S1), confirming the total screening of the applied voltage. This behavior was consistently observed throughout all the simulations in this study regardless of the frequency, ionic concentration, and system size probed. However, the relative roles played by water and ions evolve significantly with the system’s parameters.

For an imposed AC voltage at a frequency of 300 MHz and in a solution containing 210 mM NaCl (Figure 2a), the water electric dipole reacts first to the voltage, initially screening it almost perfectly until it reached the first maximum of the cycle. The ions take longer to react, resulting in a phase lag −90 < φ_Mz,ions_ < 0 in their response. As *M*_z,ions_ eventually starts to increase, the amplitude of the water dipoles decreases, resulting in the water effectively leading the voltage oscillation (0 < φ_*Mz*,wate*r*_ < 90). Halfway through the voltage period, when *V* = 0, ions create a net positive dipole moment due to their lagging response, a dipole exactly compensated for by the water molecules. This is consistent with the system preferentially using ions to screen the applied field, as in DC conditions, but with dynamics limited by the need for ions to diffuse. Water molecules then operate as a buffer by compensating for the lagging behavior of the ions.

Repeating the simulation at a higher frequency (1000 MHz, Figure 2b) or at a lower salt concentration (21 mM NaCl, Figure 2c) shows in both cases a shorter transient response, with the transient and steady-state behaviors almost indistinguishable from the onset of the voltage application. In all cases, past the short initial transient response of the system, a stable steady state is observed. The characteristics of this steady response are reproducibly dependent on the external parameters such as the AC frequency, ionic concentration, or system size. These effects are systematically explored hereafter.

### 3.2 The Impact of Frequency on the Steady-State Response

**Figure 3:**
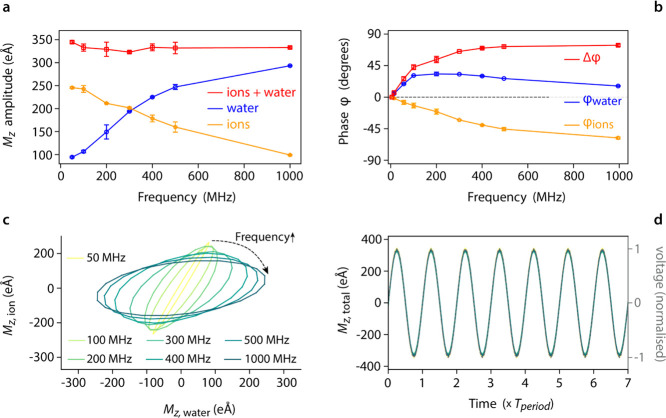
Steady-state behavior of ions and water as a function of the applied voltage frequency. The amplitude (a) and phase (b) of the ionic and water dipoles are shown as a function of the driving frequency. Plotting *M*_*z*,ion_ against *M*_*z*__,water_ offers a convenient way to visualize the same information (c), highlighting the dominating role of water at higher frequencies. The total dipole *M*_*z*__,ion_ + *M*_*z*__,water_ (vectorial sum) remains unchanged in both phase and amplitude regardless of the frequency probed (d), with the response of the system at all the frequencies indistinguishable but always canceling out the applied field. The data are shown for a 210 mM NaCl solution in a 6.88 nm gap between uncharged silica slabs.

To investigate the impact of the driving frequency on the behavior of the electrolyte, we considered a salt concentration of 210 mM with the standard 6.88 nm gap between two uncharged silica slabs. A similar analysis as presented in Figure 1 is carried out, with the amplitude and phase of the ions and water responses to the electric field shown as a function of the imposed voltage frequency (Figure 3a,b).

At lower frequencies (<300 MHz), the ions move fast enough to respond to the applied oscillating voltage, and *M*_*z*__,ion_ presents a larger magnitude than *M*_*z*__,water_ (Figure 3a). This is no longer the case at larger frequencies, with water molecules becoming the main contributor to the screening with the magnitude of *M*_*z*__,ion_ progressively decreasing. The crossover coincides with the maximum lead phase of water (Figure 3b), with the lead compensating for the ionic lag in response to the field. The phase lag of the ions increases with the frequency but never reaches a purely capacitive behavior (φ_*Mz*,ions_ → −90°) within the frequency range probed here (Figure 3c). Qualitatively, the ionic behavior can be described with an equivalent Debye circuit comprising a resistor and a capacitor in parallel.^3,13,42^ The transition frequency *f*_act_ ∼ 300 MHz between an ion-dominated and a water-dominated response approximately coincides with the expected charging time scale τ_DL_ for the electrical double layer from the electrolyte in the gap.^22^ Practically, we get *f*_act_ ∼ 1/(2 τ_DL_) because two charging/discharging events occur over a full AC cycle. Consistently, the time scale 1/*f*_act_ is also close to the so-called *actuation frequency* of the system,^3,13,43^ the frequency beyond which ions are no longer able to move fast enough to follow the applied field and start behaving capacitively. The transition from a resistive to a capacitive behavior for the ions is progressive and monotonic, with the presence of both types of behavior beyond the GHz, at least for the present conditions. In fact, test simulations revealed that a frequency of 10 GHz is needed to ensure a purely capacitive response of the ions at this salt concentration (not shown).

The amplitude and the phase of the total dipole *M*_*z*,total_ remain unchanged regardless of the frequency, always perfectly in phase with the applied field and screening it completely (Figure 3d). Because the *M*_*z*,total_ that is provided by both the water and ions remains fixed with time and frequency, the voltage between slabs remains fixed. The monotonic increase in the ionic lag and the changes in the water lead create an increase in the water–ion phase difference with frequency, which converges to ∼80° above 500 MHz (Figure 3b).

### 3.3 The Impact of Salt Concentration on the Steady-State Response

**Figure 4:**
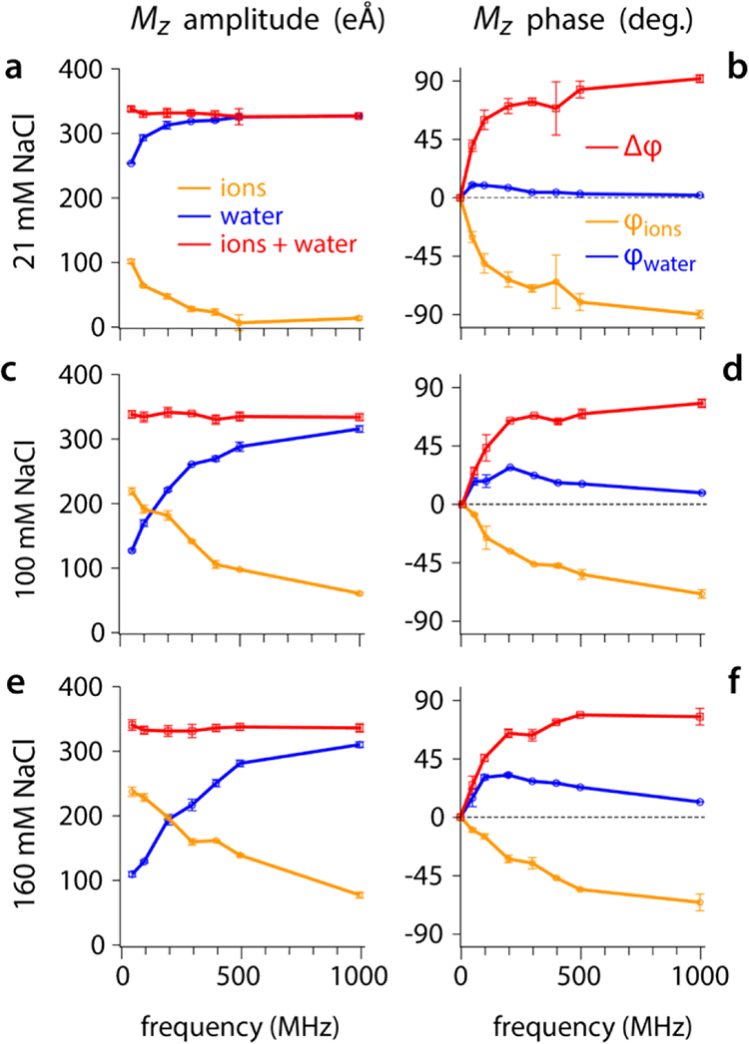
Impact of salt concentration on the electrolyte’s response to the applied AC voltage. At 21 mM (a, b), the system is always dominated by the response of water, which retains a larger *M_z_* amplitude at all frequencies. The data suggest that a crossover occurs <50 MHz with the ions screening the voltage at DC. However, the ions behave as purely capacitive at >500 MHz. Increasing the salt concentration to 100 mM (c, d) and 160 mM (e, f) shifts the crossover from an ion-dominated response at lower frequencies to a water dominated response at higher frequencies.

We next investigate the effect of the salt concentration on the electrolyte response to the AC field (Figure 4; see also Figure S2). The NaCl concentration is varied from 21 to 840 mM to cover the range typically found in biological systems and in nature. The nanogap is kept at 6.88 nm with uncharged silica slabs, but the AC frequency is varied systematically for each salt concentration, as in the previous section.

From Figure 4, it is immediately clear that the total dipole moment of the electrolyte does not change with either NaCl concentration or driving frequency, similar to the results in Figure 3. This implies that the voltage as experienced by a test charge located between the slabs does not change with either salt concentration or frequency. Qualitatively, the system behaves similarly at all salt concentrations, always following the trend observed previously at 210 mM NaCl (Figure 3). The amplitudes of *M*_*z*,ion_ and *M*_*z*,water_ respectively decrease and increase with frequency. The associated phase behavior is also consistent with the previous observations at 210 mM. However, a quantitative comparison shows significant differences between each salt concentration.

At the lowest salt concentration tested, 21 mM (Figure 4a,b), the system is dominated by the water dipole over the whole range of frequencies investigated (50–1000 MHz). No crossover is observed between *M*_*z*,ion_ and *M*_*z*,water_, but there exists a maximum in the water lead phase at the lowest frequency probed, suggesting that a crossover may exist at lower frequencies (Figure 4a). The predicted actuation frequency is around *f*_act_ ∼ 90 MHz at this salt concentration, higher than observed here from the simulations (<50 MHz). At higher frequencies (>500 MHz) the ions become completely capacitive (φ_*Mz*,ions_ ∼ −90°) and cease to play any role in opposing the applied field (Figure 4b). This is confirmed by the phase of the water, which no longer leads the AC voltage but operates perfectly in phase (φ_*Mz*,water_ ∼ 0°).

Increasing the salt concentration to 100 mM effectively shifts the balance of ions and water to higher frequencies with the dipole crossover predicted and observed around 200 MHz (Figure 4c,d). At higher frequencies, the ions tend to behave more capacitively but not fully, even at 1000 MHz. This is expected: the higher ionic concentration requires shorter diffusion distances for the ions to achieve the same net dipole. Further increasing the salt concentration to 160 mM (Figure 4e,f) follows the same trend, eventually leading to the results previously seen at 210 mM (Figure 3a,b).

### 3.4 The Impact of the Nanogap Size on the Steady-State Response

**Figure 5:**
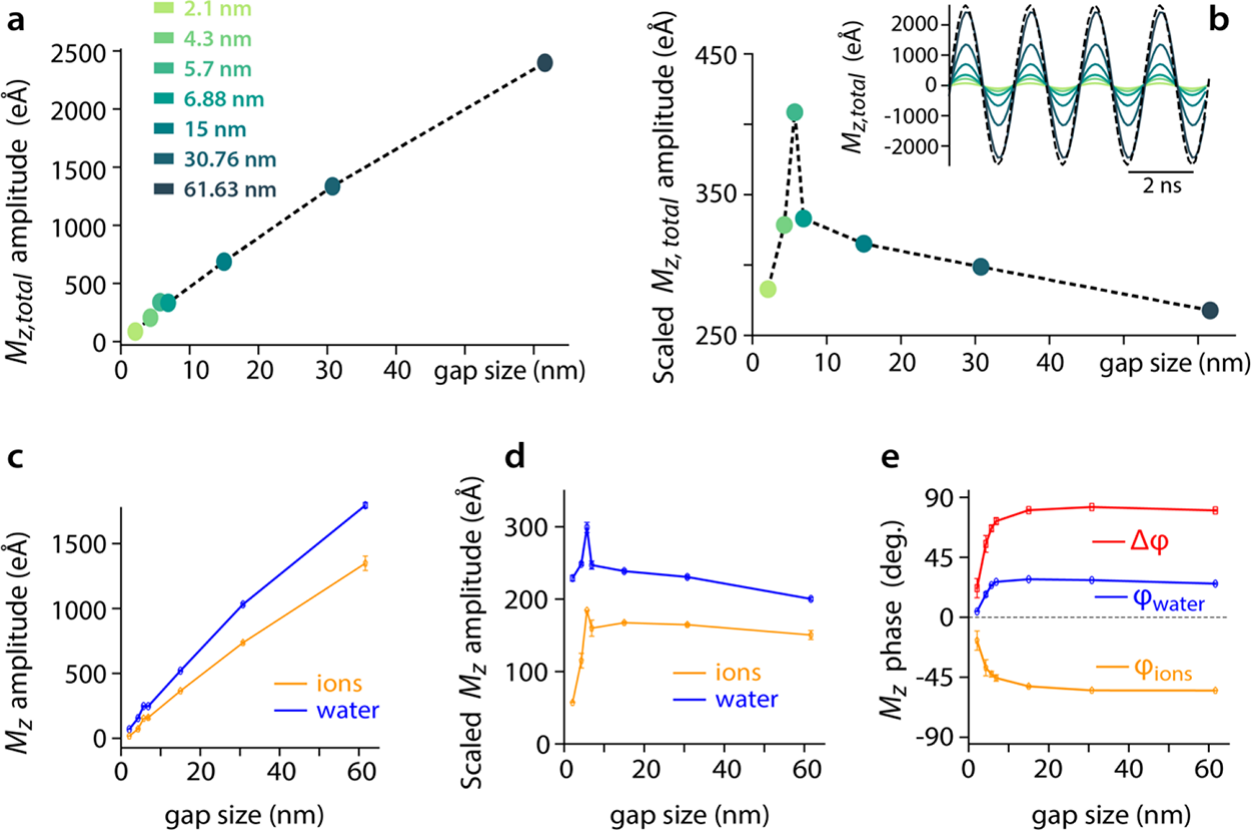
Impact of the nanogap size on the electrolyte steady-state response to an applied 500 MHz AC voltage in 210 mM NaCl. (a) *M*_*z*,total_ remains in phase with the AC field regardless of the gap size (inset), but the amplitude of *M*_*z*,total_ changes with the gap size. Simulations have been conducted at gap distances of 2.1 4.3, 5.7, 6.88, 15, 30.76, and 61.63 nm. The changes can be visualized individually for *M*_*z*,ion_ and *M*_*z*,water_ with both the amplitude (b) and phase (c) exhibiting clear differences in behavior. The driving voltage is shown dashed in panel a and normalized to the largest *M*_*z*,total_ to aid comparison.

The separation distance between the two silica slabs has a significant effect on the response of the system to the AC field (Figure 5). In keeping with our stated goal to mimic experimental settings, the magnitude of the applied voltage is kept constant for all slab separations, resulting in an electric field, the magnitude of which is, on average, inversely proportional to the gap size. The raw value of *M*_*z*,total_ is given as a function of gap size (Figure 5a and inset in b), together with scaled *M*_*z*_ values derived for each gap by the ratio of the gap size to our reference of 6.88 nm. The scaled value is proportional to the average *M*_*z*_ per water molecule and ions in the system and is intended to help ascertain the impact of the voltage per water molecule. At our reference salt concentration of 210 mM and a voltage frequency of 500 MHz, *M*_*z*,total_ remains in phase with the applied electric field regardless of the separation distance (Figure 5b, inset), but the separation distance influences the amplitude of *M*_*z*,total_ in a nonlinear manner (Figure 5a,b). *M*_*z*,total_ increases sublinearly with the gap size (Figure 5a and inset in b), an effect dependent on the salt concentration (Figure S2). This is likely due to the evolution of the water dielectric constant as a function of the gap (Figure S3) combined with added ions to the system as the gap widens. Given the fixed voltage, we anticipate *M*_*z*,total_ to converge to a constant value for larger gaps, but this convergence is not visible in the present simulations, consistent with the relatively long-range evolution of the dielectric constant (Figure S3). For gap distances shorter than 5.7 nm, the amplitude of the total dipole is lower than the corresponding value for the separation distance of 6.88 for which, according to our DC simulations, the *M*_*z*,total_ is enough for screening the whole field. This is clearly visible in the scaled *M*_*z*,total_ value (Figure 5b). This indicates an underscreening for shorter separation distances with the effect decreasing as the gap size widens. The amplitude and phase of the water and ionic responses are individually influenced by the plate separation distance (Figure 5c–e). The change in the water dipole and ion dipole amplitudes follows the same trend as the total dipole moment (Figure 5c,d). In contrast, the lag in the ionic response increases with the separation distance owing to the need for ions to diffuse in longer distances, which requires longer times (Figure 5e). Consistently, the ionic lag is compensated for by an increased lead in the water response to the applied field (Figure 5e). The maximum in the scaled net dipole amplitude, observed around 5.7 nm, is more marked for water than for the ions.

The molecular origins of this contrasting behavior between gap sizes below and above 5.7 nm are not immediately obvious from the simulations, but the results suggest two different screening regimes. Previous work on a similar silica system under DC voltages^35^ revealed significant interfacial effects on the behavior of water in gaps as large as 7 nm, with an average water dielectric constant maximized at a salt concentration of 210 mM. Here, the two regimes could be explained as interface-dominated below 5.7 nm with a significant effect of the surface on the behavior of water and ions, turning to bulk solution-dominated for larger distances. Repeating the simulations at different ionic concentrations (21 and 840 mM; Figure S2) tends to support this hypothesis. At 21 mM, the behavior of the system’s response is dominated by water due to the low concentration of ions that have to diffuse larger distances. Consistently, water’s response is in phase with the applied field, whereas the ions behave capacitively at all gap sizes. In contrast, at 840 mM, a smooth transition between the two regimes is visible, with ions playing a dominating role at all but the smallest gap distance due to their high concentration that allows them to rearrange rapidly in response to the field. The phase of the ions is no longer purely capacitive but remains constant for all gap distances. The phase of the water increases to positive values as the gap initially grows, suggesting interfacial effects to still be at play regardless of the salt concentration.^35^ The overall increase of the amplitude of *M*_*z*,ion_ (Figure S2a–f) and *M*_*z*,total_ at larger gap sizes simply reflects a better ability for the ions to screen the applied field at higher concentrations. The behavior of the system at 210 mM (Figure 5) falls into an intermediate situation with both water and ions playing a comparable role in the screening. The water leads the phase and the ions lag, but both evolve from a phase close to zero at the smallest gap size to a near constant value in the “bulk” regime. The sharpness of the transition observed in Figure 5 is specific to 210 mM, likely due to some nonlinearities in the effects at play close to the transition between the two regimes. However, the limited resolution in gap sizes available in this study precludes a firm conclusion.

Considering the water dielectric constant and the density profiles of water molecules and ions for different gap sizes can also help with the interpretation (Figure S3). The dielectric constant can be used as a proxy for molecular alignment of the water because it reduces with improving alignment. Consistently, in the regime interpreted as interface-dominated, the dielectric constant is lower than for the unconfined system, whereas in the bulk regime, the dielectric constant recovers to the unconfined value (Figure S3a). The salt density distribution also shows regions of excluded ions near the interface (Figure S3b), an effect that relatively affects a larger portion of the gap size for the lower separation distance when compared with larger separations (Figure S3c).

### 3.5 The Impact of the Slab Material and Charge on the Steady-State Response

In the previous sections, we always considered an aqueous NaCl solution placed between two neutral silica slabs. In this section, we compare the results obtained with neutral silica slabs to a system composed of one charged silica slab and one gold slab, as a function of the applied AC frequency, with a nanogap size of 6.88 nm in both cases. This choice of material is motivated by its frequent use in nanoscale experimental measurements over this frequency range.^3,18,19,21,23,27^ Gold is often used as a conducting nanoelectrode either in devices^3,18^ or as a conducting scanning probe.^23^ Silica is a common hydrophilic substrate, with thin thermal oxide layers often used as blocking electrodes.^19,23,27^ However, unless particular care is taken to adjust the pH of the electrolyte so as to reach silica’s point of zero charge, the silica surface is negatively charged in most situations.

**Figure 6:**
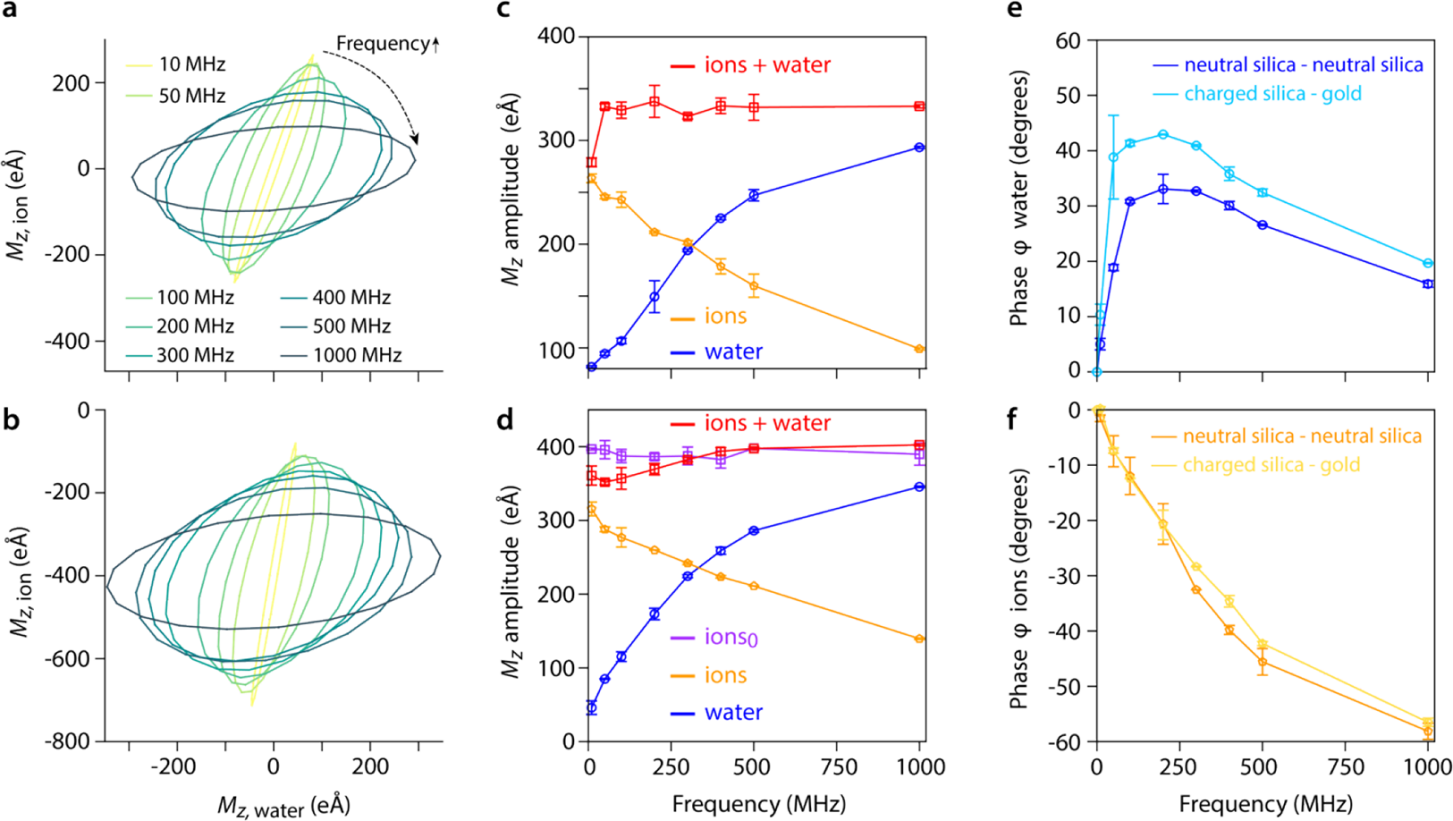
Impact of the slab material and surface charge on the response of the system. Plotting *M*_*z*,ion_ vs *M*_*z*,water_ for the neutral–neutral silica system (a) and the charged silica–gold system (b) shows a similar evolution with frequency. A DC offset is visible for *M*_*z*,ion_ in the charged silica–gold system, reflecting the presence of static surface charges on the silica. This offset is constant and does not evolve with the frequency. The amplitudes of *M*_*z*,ion_, *M*_*z*,water_, and *M*_*z*,total_ are shown as a function of frequency for the neutral–neutral silica system (c) and the charged silica–gold system (d). In the latter case, the ion has been separated into its invariant DC component (ion_0_) and its AC response (ion). *M*_*z*,total_ slightly increases with frequency, unlike in the neutral system. The associated phase evolution shows comparatively the evolution of water (e) and ions (f) for both systems, with a significant difference only visible for water. All the simulations are run at 210 mM NaCl and with a 6.88 nm gap size.

To ascertain the comparability of the charged silica–gold system with our neutral silica reference, we conducted preliminary simulations of a system composed of two charged silica surfaces. The results showed that in the stationary regime, the counterions in close vicinity to the surface stop responding to the field due to their interaction with the charges on the silica. As a consequence, the system effectively behaved like a system made of two neutral silica slabs. In fact, comparison of the charged silica–gold system with our neutral silica reference shows a very similar behavior in both cases for water and ions (Figure 6a,b). However, the absolute value of *M*_*z*,ion_ is centered around 400 *e*Å in the system with charged silica (unlike zero for neutral silica). This simply reflects the static component of the ionic response to silica’s surface charge, effectively acting as a DC offset to the ionic behavior. The overall AC behavior remains largely unchanged (Figure 6c,d), but subtle differences arise. For example, the total dipole *M*_*z*,total_ is largely frequency-independent for the neutral silica–neutral silica system (Figure 6c) but shows a slight increase with the frequency for the charged silica–gold system (Figure 6d), implying a lower voltage across the system at higher frequencies. The phase lag in the ionic response is also similar in both systems (Figure 6e,f), but the lead in the water response depends on the slab material: water consistently has a larger lead (up to 10°) in the charged silica–gold system. Overall, these results demonstrate a subtle but clear impact of the interfacial material on the response of the electrolyte to AC and DC fields both in magnitude and in phase.

## 4 Discussion

This study investigates systematically the response of ions and water molecules to an applied AC field across a nanogap as a function of the field frequency, ionic concentration, nanogap size, and the nature of the confining surfaces. Generally, the findings for the observed ionic behavior are in line with the theory^3,13,43^ and with previous studies operating in the same frequency range.^19,23,43^ The ability of ions to follow and screen the applied field is limited by their diffusion through the solution, rendering them less effective at lower concentrations or higher frequencies. This can be quantified by the phase lag of the net dipole formed by the cation and anion distribution throughout the system, assuming a behavior analogous to a resistive–capacitive circuit. In this framework, the ions exhibit a resistive behavior when diffusing through the solution and a capacitive behavior when no longer able follow the applied field. The frequency and ionic concentrations marking the transition from a resistive to purely capacitive behaviour can be described relatively well by continuum models.^9,10^ These observations assume that the system operates stably, linearly, and has reached a steady state under the imposed AC potential. The transients from a state where no potential is applied to a steady state could also be visualized, showing a rapid (typically less than half a period) evolution toward the steady state.

The present study highlights the role of water molecules in opposing the field, with water consistently leading the imposed AC voltage. This is due to water compensating for the lag in response to the ions, as evidenced in the transients. This results in a total response of the electrolyte perfectly in phase with the applied voltage. Consistently, the phase lead of the net dipole formed by water molecules tends to zero close to DC or when the ions behave capacitively. This results in the water showing a maximum lead at specific frequencies that depend on the ionic concentration and tend to coincide with the ions’ transition from resistive to capacitive behavior. In the resistive–capacitive analogy used to describe the behavior of the ions, water would effectively act as an inductor, potentially inducing some resonances in the overall response of the electrolyte to the field. It is, however, not obvious whether applying this circuit analogy to water makes sense because its “inductive” behavior is a result of that of the ions. We note that a previous experimental study based on atomic force microscopy reported resonances in the measured dielectric force that scaled with the static dielectric constant of the solvent and that where reduced when increasing the ionic concentration of the solution.^23^ While interesting in light of the present simulations, it is important to keep in mind that other explanations for these resonances, such as imperfection in the experimental setup, are also possible.

The present simulations also highlight the importance of interfacial effects, where water and ions interact directly with the confined solid surfaces. Reducing the gap size for a set applied voltage leads to an increase in the electric field, which, in turn, should result in a higher magnitude of the electrolyte response to the applied voltage. However, for shorter separation distances, the trend is counterintuitively reversed due to interfacial effects. We speculate that the molecular origins of the effect is twofold: confinement and ionic mobility. According to the Poisson–Boltzmann equation, the interface voltage reaches the bulk value at ∼5 times the Debye length,^9^ here approximately the distance from the center of the simulation box to each of the silica plates. This implies that the system effectively behaves as confined at this distance and concentration. The interaction of the water molecules with the silica surfaces is also likely to play a role as part of the confinement behavior. Additionally, the response of the ions to the external field can vary from a simple position exchange with neighbors at higher concentrations to the diffusion over relatively large distances at lower concentrations. This implies that the diffusion mechanism becomes more prominent at a specific separation distance. Interestingly, the activation of the diffusion mechanism and switching from confined to nonconfined system appear to create a sharp peak in the *M*_*z*,total_.

To investigate interfacial effects more directly, we replaced the two neutral silica slabs by one charged silica surface and one gold surface. Aside from the relevance of the materials for science and technology, this offers a direct point of comparison with neutral silica, with the simulations conducted at the same gap size of 6.88 nm, past the maximum dipolar response discussed above. The results show that although the general behavior of the electrolyte is similar in both cases, clear differences arise. First, the presence of a charged silica slab induces a static shift in the ionic dipole, indicating counterions being strongly adsorbed to the slab and no longer responding to the AC voltage. These ions can effectively be seen as part of the charge silica slab that then becomes neutral. Given the magnitude of the voltages needed to remove these ions (typically >50 V, from preliminary simulations), it is reasonable to assume the silica surfaces to effectively behave as neutral silica in first approximation, both in theoretical^35^ and in experimental^23,27,43^ studies involving electric fields through an aqueous electrolyte. Second, the presence of a gold 111 surface appears sufficient to affect the average behavior of the water molecules across the gap, with an increased phase lead on the driving voltage and marginally lower response at lower frequencies. This could be explained by the crystalline nature of the gold surface, potentially reducing the configuration entropy of the water molecules and increasing the time required for rearrangement in the first solvation layer of the surface. Previous simulations done by Limmer et al.^44^ and Willard et al.^45^ showed that the crystal structure of solid surfaces affects the time scale for rearrangement of water molecules, spanning single to several tens of nanoseconds with even longer times in some cases. This is also consistent with the fact that the frequency with maximum water lead shifts toward lower values when gold is present, suggesting that not only ions but also slower interfacial water is at play. The fact that the effect is measurable in a ∼7 nm gap where only one surface is crystalline suggests that it may play an important role for nanoconfined systems. Such systems are ubiquitous in nature^46,47^ and in technological applications,^48,49^ including under applied electrical potentials.^4,5^

## 5 Conclusions

In this study, we use atomistic simulations to investigate the behavior of water and dissolved NaCl ions into a nanogap when under an external AC voltage. We focus on radio frequencies (10 MHz–1000 MHz) where the ability of the ions to diffuse tends to become the limiting factor for opposing the applied field. The simulations aim to replicate the main features of recent experimental developments in the field of dielectric force microscopy in electrolyte solutions, quantifying the interplay between ionic mobility, water reorientation, and interfacial effects at the surface of the immersed solids. The results reproduce the well-established resistive–capacitive behavior of the electrolyte with ions dominating the response to the applied field at lower frequencies while only water can react at higher frequencies. However, the total screening remained largely independent of the frequency with water systematically compensating for any ionic lag, except when prevented to do so by interfacial interactions with the solid. As a result, water often leads to applied voltage.

This work sheds some light on the molecular response of aqueous electrolytes to applied fields in nanogaps, in particular, the interfacial aspects that cannot be easily captured by continuum descriptions of the liquid. We anticipate our findings to be of interest to the scanning probe community as well as for the development of nanosensors working in solution.

## Data Availability

The data that support this study including all the simulation files are freely available from the Durham University online repository at https://collections.durham.ac.uk/files/r2k3569440p (doi:10.15128/r2k3569440p).

## Supporting Information Available

Summary of all the simulations run (Table S1) and additional results and controls (Figures S1–S3) (PDF)
