# Supplementary material for: Electrified Nanogaps under an AC Field: A Molecular Dynamics Study
Source: J Phys Chem C Nanomater Interfaces. 2024 Nov 29;128(49):21050–9. doi: 10.1021/acs.jpcc.4c05105 (PMC11648076; doi:10.1021/acs.jpcc.4c05105)
Supplement: Supplementary file 1 — jp4c05105_si_001.pdf [file jp4c05105_si_001.pdf]

Supplementary information

Electrified Nanogaps under AC Field: A Molecular Dynamics Study

Mahdi Tavakol[1,*,†] Alexander Newbold[1] and Kislon Voïtchovsky[1,*]

1. Physics Department, Durham University, Durham DH1 3LE, UK

[†]current address: Department of Engineering Science, University of Oxford, Oxford, OX1 3PJ, UK

*corresponding authors: mahditavakol90@gmail.com , kislon.voitchovsky@durham.ac.uk

Table of content of the Supplementary Online Information

- Table S1: Summary of the different simulations carried out for this study
- Figure S1: Response of the system to a DC field
- Figure S2: Response of the system to a 500 MHz AC field for different salt concentrations and gap sizes.
- Figure S3: Impact of the silica separation distance on the water and ions' response to the system under a 500MHz AC voltage in 210mM NaCl.

Table S1 – summary of the different simulations carried out for this study

Run ID	Sim set	# of sims	Slab Materials	Vmax (V)	AC Freq (MHz)	Gap (nm)	Ion conc. (mM)	Cumulat. time (ns)	Water Molecules (#)	Na^+ (#)	Cl^- (#)
276	1	14	Neutral silicas	14.14	300	6.88	210	90	~6900	24	24
279	2	12	Neutral silicas	14.14	1000	6.88	210	70			
344	3	11	Neutral silicas	14.14	300	6.88	21	50	~7000	2	2
268	4	2	Neutral silicas	14.14	50	6.88	210	70	~6900	24	24
269	5	2	Neutral silicas	14.14	100	6.88	210	40			
270	7	2	Neutral silicas	14.14	200	6.88	210	25			
277	9	2	Neutral silicas	14.14	400	6.88	210	90			
272	10	2	Neutral silicas	14.14	500	6.88	210	90			
341	11	2	Neutral silicas	14.14	50	6.88	21	70	~7000	2	2
342	12	2	Neutral silicas	14.14	100	6.88	21	70			
343	13	2	Neutral silicas	14.14	200	6.88	21	70			
344	14	2	Neutral silicas	14.14	300	6.88	21	70			
345	15	2	Neutral silicas	14.14	400	6.88	21	35			
346	16	2	Neutral silicas	14.14	500	6.88	21	70			
347	17	2	Neutral silicas	14.14	1000	6.88	21	20			
381	18	2	Neutral silicas	14.14	50	6.88	100	90	~7000	12	12
383	19	2	Neutral silicas	14.14	100	6.88	100	70			
384	20	2	Neutral silicas	14.14	200	6.88	100	60			
385	21	2	Neutral silicas	14.14	300	6.88	100	40			
388	22	2	Neutral silicas	14.14	400	6.88	100	35			
389	23	2	Neutral silicas	14.14	500	6.88	100	30			
390	24	2	Neutral silicas	14.14	1000	6.88	100	20			
391	25	2	Neutral silicas	14.14	50	6.88	160	90	~7000	18	18
392	26	2	Neutral silicas	14.14	100	6.88	160	60			
393	27	2	Neutral silicas	14.14	200	6.88	160	60			
394	28	2	Neutral silicas	14.14	300	6.88	160	40			
395	29	2	Neutral silicas	14.14	400	6.88	160	35			
396	30	2	Neutral silicas	14.14	500	6.88	160	30			
397	31	2	Neutral silicas	14.14	1000	6.88	160	20			
288	32	2	Neutral silicas	14.14	500	2.10	210	50	~1700	6	6
312	33	2	Neutral silicas	14.14	500	4.30	210	90	~3500	12	12
313	34	2	Neutral silicas	14.14	500	5.70	210	50	~5200	18	18
273	35	2	Neutral silicas	14.14	500	15.00	210	50	~14800	48	48

281	36	2	Neutral silicas	14.14	500	30.76	210	50	~30600	96	96
307	37	2	Neutral silicas	14.14	500	61.63	210	50	~61200	192	192
278	38	2	Gold – Charged Silica	14.14	50	6.88	210	90	~7100	40	24
284	39	2	Gold – Charged Silica	14.14	100	6.88	210	90			
285	40	2	Gold – Charged Silica	14.14	200	6.88	210	90			
286	41	2	Gold – Charged Silica	14.14	300	6.88	210	90			
287	42	2	Gold – Charged Silica	14.14	400	6.88	210	90			
275	43	2	Gold – Charged Silica	14.14	500	6.88	210	90			
280	44	2	Gold – Charged Silica	14.14	1000	6.88	210	70			
313	45	2	Neutral silicas	14.14	500	5.70	21	50	~5200	2	2
266	46	2	Neutral silicas	14.14	500	15.00	21	20	~14800	4	4
267	47	2	Neutral silicas	14.14	500	30.76	21	20	~30600	8	8
317	48	2	Neutral silicas	14.14	500	61.63	21	50	~61200	16	16
350	49	2	Neutral silicas	14.14	500	2.10	840	90	~1700	24	24
351	50	2	Neutral silicas	14.14	500	4.30	840	90	~3500	48	48
357	51	2	Neutral silicas	14.14	500	5.70	840	50	~5200	72	72
353	52	2	Neutral silicas	14.14	500	6.88	840	90	~6900	96	96
354	53	2	Neutral silicas	14.14	500	15.00	840	50	~14400	192	192
355	54	2	Neutral silicas	14.14	500	30.76	840	50	~29700	384	384
358	55	2	Neutral silicas	14.14	500	61.63	840	20	~58100	768	768
338	56	2	Gold – Charged Silica	14.14	500	4.30	210	50	~3500	28	12
339	57	2	Gold – Charged Silica	14.14	500	5.70	210	50	~5200	34	18
340	58	2	Gold – Charged Silica	14.14	500	6.88	210	90	~7100	40	24
335	59	2	Gold – Charged Silica	14.14	500	15.00	210	90	~14800	64	48
336	60	2	Gold – Charged Silica	14.14	500	30.76	210	90	~30600	112	96
337	61	2	Gold – Charged Silica	14.14	500	61.63	210	20	~61200	208	192
400	62	2	Neutral Silicas	14.14	10	6.88	210	150	~6900	24	24
340	63	3	Gold – Charged Silica	14.14	10	6.88	210	100	~7100	40	24
349	64	2	Neutral Silicas	14.14	10	6.88	21	50	~7000	2	2
413	65	2	Neutral Silicas	14.14	0	6.88	210	50	~6900	24	24
Total Simulation time (μs)								10.09 μs			

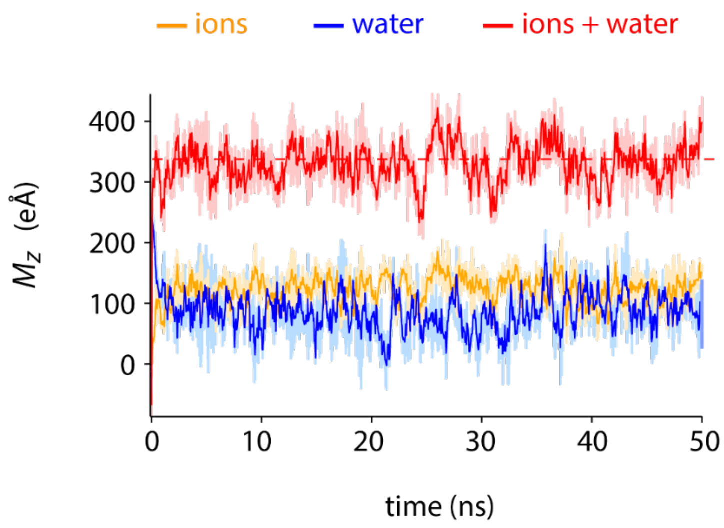

Figure S1 – Behaviour of the system under of DC voltage. After a short transient (<1 ns), the system equilibrates yielding constant values for $M_{z,ion}$, $M_{z,water}$ and $M_{z,total}$, aside for statistical fluctuations. The applied voltage is of 14.14 V, the NaCl concentration is 0.21 M and the gap size is 6.88 nm. The shaded area around the solid curves represents two standard deviations (two simulation runs of 50 ns each).

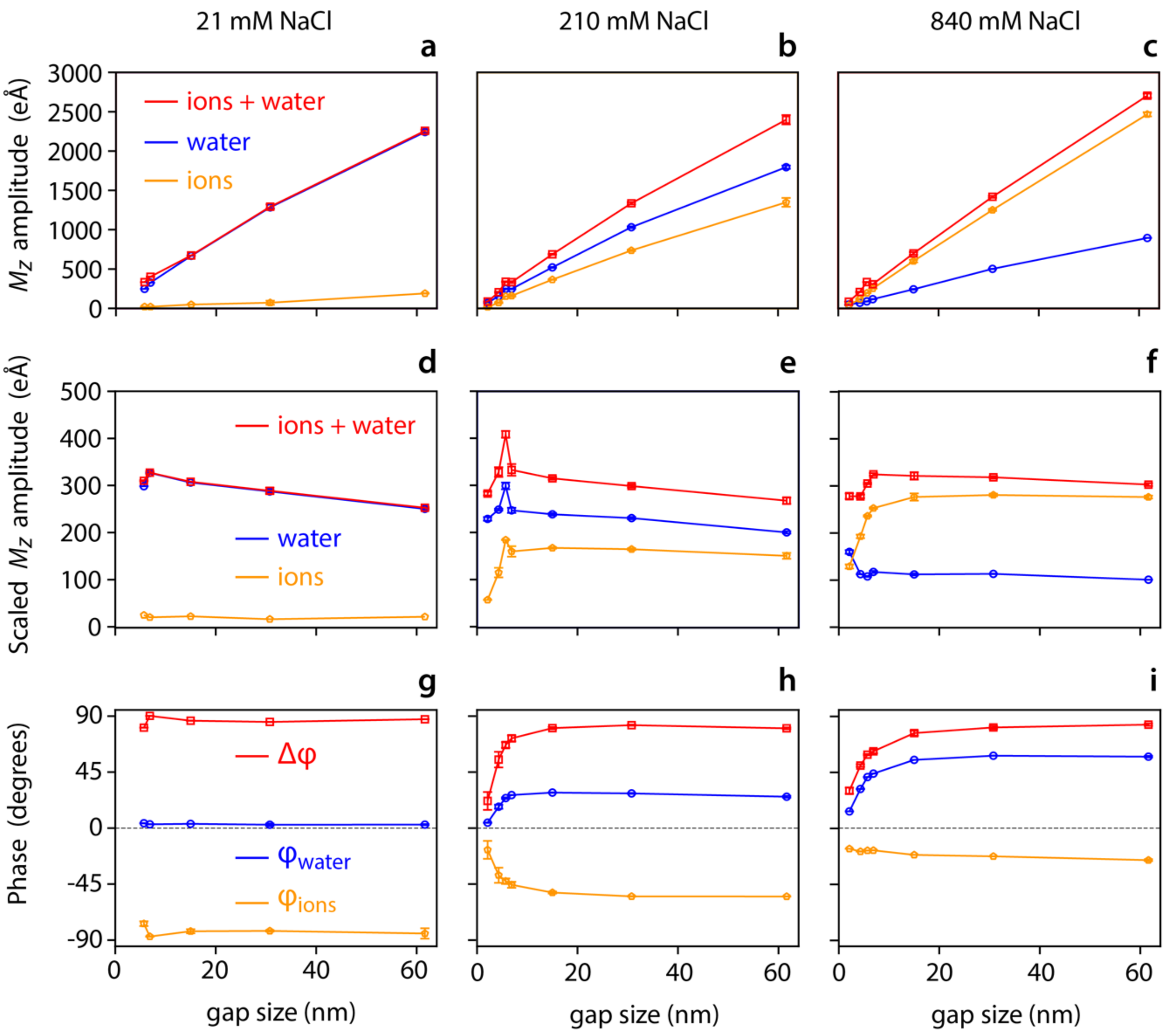

Figure S2 – Impact of the NaCl concentration on the response of water and ions to the AC voltage (500MHz) as function of the silica-silica separation distances. The amplitude of $M_{z,ion}$, $M_{z,water}$ and $M_{z,total}$, are shown for aqueous solutions containing 21 mM NaCl (a), 210 mM NaCl (b), and 840 mM NaCl (c). The sames values normalised by the gap size are given in (d), (e) and (f) respectively while the associated phase is given in (g), (h) and (i) respectively.

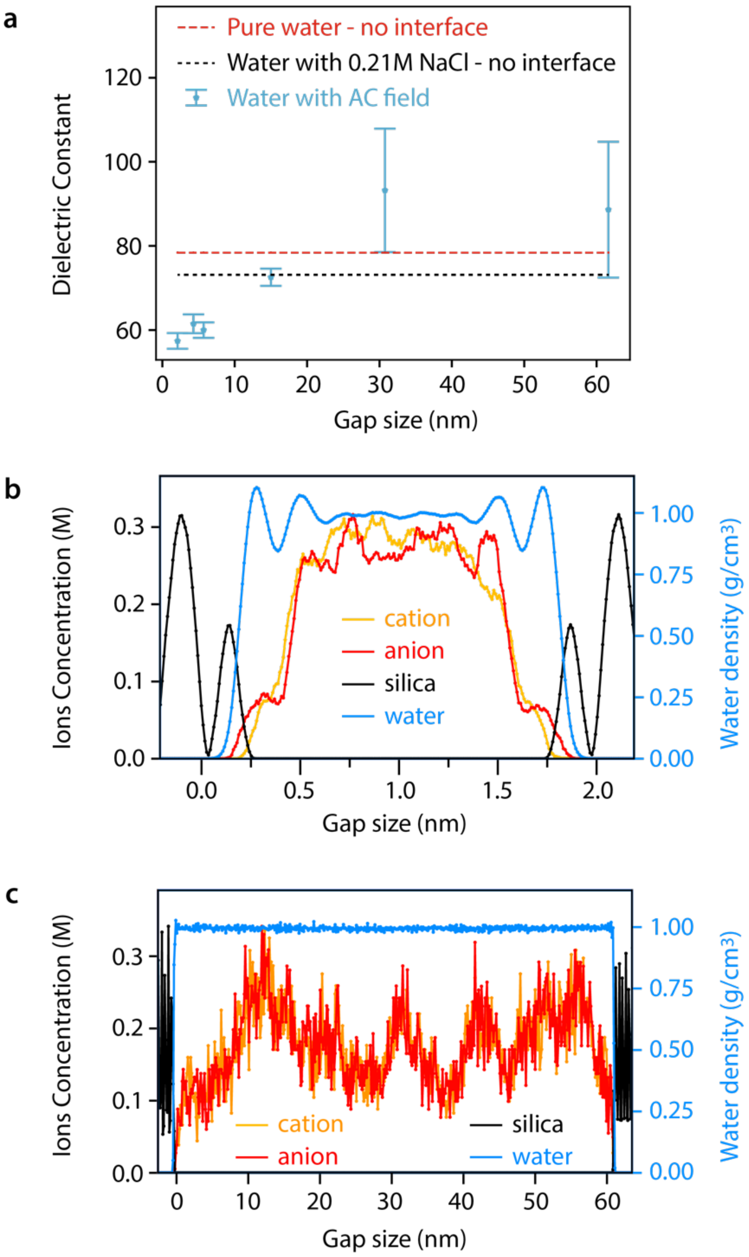

Figure S3 – Impact of the silica separation distance on the water and ions' response to the system under *a* 500MHz AC voltage in 210mM NaCl. (a) The variation in the dielectric constant with distance alongside with the dielectric constants for unconfined pure and 210mM NaCl solvated water. Ion and water density distribution between two silicas for the separation distance of (b) 2.1nm and (c) 61.63nm.
